# Prediction of Drug–Target Interactions by Combining Dual-Tree Complex Wavelet Transform with Ensemble Learning Method

**DOI:** 10.3390/molecules26175359

**Published:** 2021-09-03

**Authors:** Jie Pan, Li-Ping Li, Zhu-Hong You, Chang-Qing Yu, Zhong-Hao Ren, Yao Chen

**Affiliations:** School of Information Engineering, Xijing University, Xi’an 710123, China; JiePan960930@gmail.com (J.P.); zhuhongyou@gmail.com (Z.-H.Y.); xaycq@163.com (C.-Q.Y.); zhonghaoren98@gmail.com (Z.-H.R.); chenyao98cy@gmail.com (Y.C.)

**Keywords:** drug–target interaction, dual-tree complex wavelet transform, position-specific scoring matrix, rotation forest

## Abstract

Identification of drug–target interactions (DTIs) is vital for drug discovery. However, traditional biological approaches have some unavoidable shortcomings, such as being time consuming and expensive. Therefore, there is an urgent need to develop novel and effective computational methods to predict DTIs in order to shorten the development cycles of new drugs. In this study, we present a novel computational approach to identify DTIs, which uses protein sequence information and the dual-tree complex wavelet transform (DTCWT). More specifically, a position-specific scoring matrix (PSSM) was performed on the target protein sequence to obtain its evolutionary information. Then, DTCWT was used to extract representative features from the PSSM, which were then combined with the drug fingerprint features to form the feature descriptors. Finally, these descriptors were sent to the Rotation Forest (RoF) model for classification. A 5-fold cross validation (CV) was adopted on four datasets (Enzyme, Ion Channel, GPCRs (G-protein-coupled receptors), and NRs (Nuclear Receptors)) to validate the proposed model; our method yielded high average accuracies of 89.21%, 85.49%, 81.02%, and 74.44%, respectively. To further verify the performance of our model, we compared the RoF classifier with two state-of-the-art algorithms: the support vector machine (SVM) and the k-nearest neighbor (KNN) classifier. We also compared it with some other published methods. Moreover, the prediction results for the independent dataset further indicated that our method is effective for predicting potential DTIs. Thus, we believe that our method is suitable for facilitating drug discovery and development.

## 1. Introduction

Detecting the interactions between compounds (drugs, molecules, ligands) and proteins (targets) is one of the most active parts of the genomic drug development field, as it plays a critical role during the discovery of novel drug candidates [1]. According to statistics from the US Food and Drug Administration (FDA), it takes at least billions of dollars to develop a new drug [2]. However, only a few drug candidates will be allowed to enter the market, as most of them fail in clinical trials and show uncertain side effects [3]. Furthermore, some studies have reported that the interactions between target proteins and drugs have a significant impact on the toxic side-effects of the drug candidates [4]. This makes the study of drug–target interactions (DTIs) very useful for detecting the toxicity of candidate drugs. Over the past few years, numerous experimental approaches have been introduced to identify DTIs, but few of them have been tested and detected as interactive [5,6]. In addition, these traditional experimental-based methods need to address the problem of high false-positive and false-negative rates [7]. For these reasons, there was a strong demand for the development of novel computational approaches to shorten the drug development cycle and reduce the time taken to detect drug–target pairs [8].

With the rapid increase in publicly available chemical and biological data, various types of related databases based on the relationships between drugs and proteins (targets) have been established, such as DrugBank [9], KEGG [10], TTD [11], and SuperTarget & Matador [12]. These public databases store a large amount of DTI information, and it is essential for researchers to develop novel and robust computational methods for detecting potential DTIs on a genome-wide scale.

To date, many computational approaches combining biological information and descriptor information have been used, such as Docking simulation [13,14], ligand-based methods [15], and literature text mining methods [16], which can be employed to identify drug–target interactions. However, these methods also have some inevitable limitations. Docking simulation is an effective molecular model which can use the dynamic simulation to predict the positive interactions between drug molecules and target proteins. It usually needs the information about the 3D structural data of the targets, a requirement that is difficult to meet because this information is only available for a small fraction of all proteins. Text mining is a special method in molecular biology, which is usually used to reveal the associations between proteins or genes and their functional relationships from text documents. It uses keywords to detect potential drug–target protein interactions, but it is hard to make good use of them. For these drawbacks, it is more practical to develop novel computational models to identify DTIs without the need for information about ligands and 3D target structures.

Recently, various approaches have been reported for detecting novel DTIs. Yamanishi et al. [17] developed a new statistical method that uses genomic sequence information and chemical structure to predict unknown DTI networks. Wang et al. [18] reported a computational model, which utilized a stacked auto encoder based on deep learning that can effectively extract raw data information to identify drug–target interactions. Hao et al. [19] introduced a useful algorithm, called dual-network integrated logistic matrix factorization (DNILMF), which consists of four steps to detect potential drug–target interactions. Wen et al. [20] suggested a deep-learning based algorithm called DeepDTIs. DeepDTIs utilized unsupervised pretraining to abstract representations from raw input descriptors. It can be applied to detect whether a new target interacts with some existing drugs. Ezzat et al. [21] presented a framework that combined feature dimensionality reduction and the ensemble learning model for predicting DTIs. Huang et al. [22] developed a method called MolTrans (Molecular Interaction Transformer) to predict DTIs that combined the interaction modeling module and sub-structural pattern mining algorithm. Zhang et al. [23] developed a method called SPVes that combined SMILES2Vec and ProtVec to convert SMILES strings of drug compounds and sequences of target proteins as feature vectors to predict DTIs. Wang et al. [24] built a heterogeneous drug–target graph to detect DTIs. It used known DTIs, drug–drug, and target–target similarities. Redkar et al. [25] used dipeptide composition and drugs with a molecular descriptor to encode the target protein sequence, and then a machine learning method that combined wrapper feature extraction and the synthetic minority oversampling technique (SMOTE) was adopted to predict DTIs. Although these methods have accelerated discoveries concerning drug–target interactions, there is still room for improvement.

In this study, we present a computational approach to identify potential DTIs based on the information of chemical fingerprints and target protein sequences. The prediction process is divided into three stages. Firstly, the target protein sequences were transformed into position-specific score matrices (PSSMs) to obtain their evolutionary information. Secondly, an effective feature extraction method, dual-tree complex wavelet transform (DTCWT), was performed to extract feature vectors from the PSSMs. Finally, we combined the drug molecule fingerprint information with these vectors to construct feature descriptors and fed them into the Rotation Forest (RoF) classifier. From the voting results of these decision trees, we can observe whether these drugs and target proteins are most likely to interact with each other. To verify the predictive ability of the proposed method, we applied a 5-fold cross-validation (CV) on four benchmark datasets: Enzyme, Ion Channel, GPCRs (G-protein-coupled receptors), and NRs (Nuclear Receptors). Furthermore, we compared the predictive performance of the proposed model with state-of-art SVM and KNN classifier and applied our method on an independent dataset. The comprehensive results demonstrated that our approach is efficient and reliable for predicting potential DTIs.

## 2. Results

### 2.1. Evaluation Metrics

**Figure 1:**
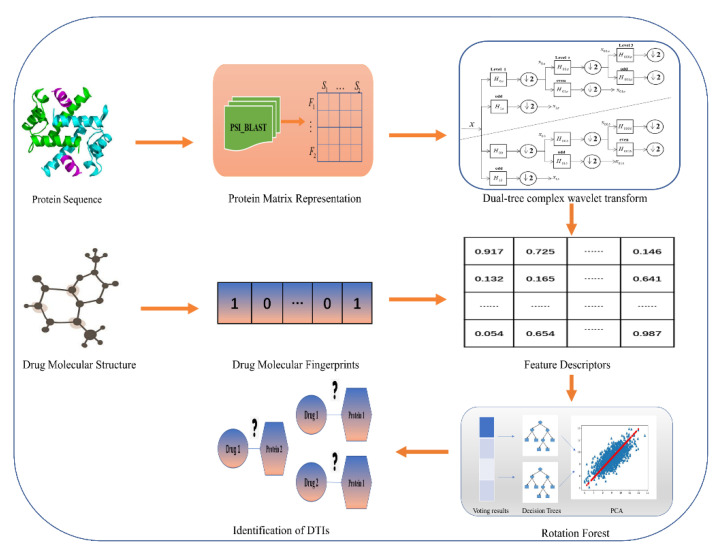
Framework of the proposed method to predict DTIs.

In this work, to access the predictive capacity of the proposed approach, we employed four evaluation metrics: accuracy (ACC.), precision (PR.), sensitivity (Sen.), and the Matthews correlation coefficient (MCC). These conventional evaluation indicators can be defined as follows:

(1) $$ACC.=\frac{TN+TP}{TP+TN+FP+FN}$$

(2) $$PR.=\frac{TP}{TP+FP}$$

(3) $$Sen.=\frac{TP}{FN+TP}$$

(4) $$MCC=\frac{TP\cdot TN-FN\cdot FP}{\sqrt{\left(TN+FN)(FN+TP)(TP+FP)(FP+TN\right)}}$$

where true positive (*TP*) represents the number of interacting drug–target pairs predicted correctly, false positive (*FP*) represents the number of non-interacting pairs predicted to be interacting, true negative (*TN*) represents the number of non-interacting pairs predicted correctly, and false negative (*FN*) represents the number of interacting pairs predicted to be non-interacting. Receiver Operating Characteristic (ROC) curves [26] were plotted based on these parameters, and the area under the ROC curves (AUC) was calculated to summarize the ROC curves numerically. In this way, we were able to provide a more comprehensive measure than other evaluation metrics. The flowchart of the proposed approach for identifying potential DTIs is shown in Figure 1.

### 2.2. Parameter Discussion

**Figure 2:**
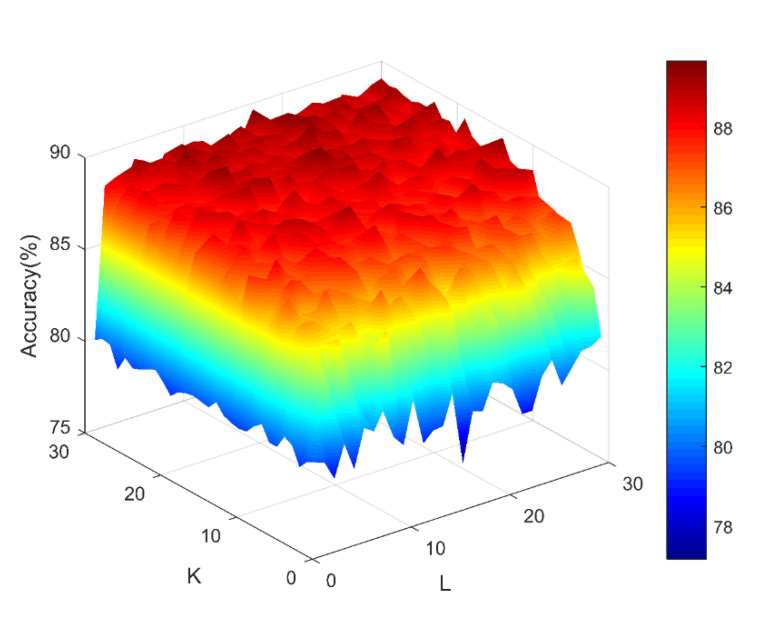
Surface of accuracy results formed by parameters K and L.

To achieve better prediction results, it is important to optimize the best parameters K and L for the Rotation Forest (RoF) model. Here, K represents the number of feature subsets and L represents the total number of decision trees in the RoF classifier. In this part, we used the grid research for the optimal parameters of RoF. Figure 2 shows the accuracy surface, which was generated by the RoF model and influenced by the parameters K and L. It can be observed that when K = 30 and L = 17, the model obtained the best predictive performance. In this work, we set the K value and the L value to be 30 and 17, respectively.

### 2.3. Performance Evaluations on Four Golden Standard Datasets

To further test the reliability of our method and avoid over-fitting, we performed 5-fold cross-validation (CV) on four datasets (Enzyme, Ion Channel, GPCRs, and NRs). More specifically, the DTI datasets were split into five parts; four of them were used as the training set and the remaining one was employed to test the model. In this way, the *CV* process was repeated for five rounds to generate five models. For the sake of consistency, all the parameters of these experiments have been kept identical in this study. Table 1, Table 2, Table 3 and Table 4 present the results of the proposed model when adopting the 5-fold CV on four collection datasets.

**Figure 3:**
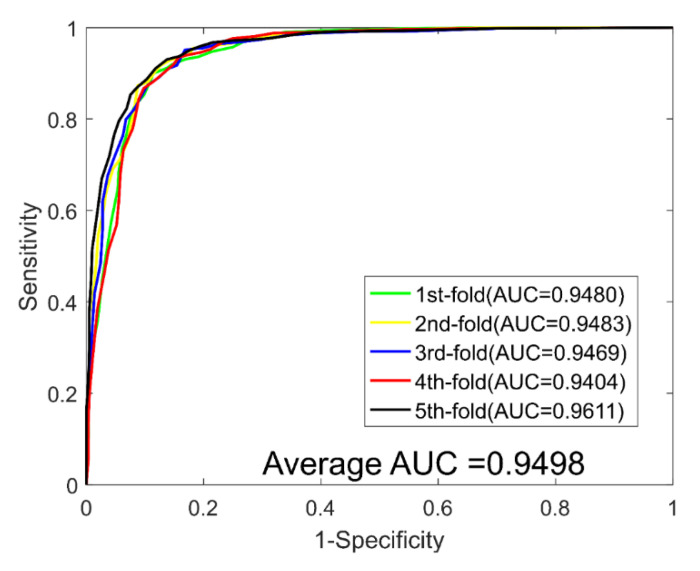
ROC curves yielded for RoF on the Enzyme dataset.

**Figure 4:**
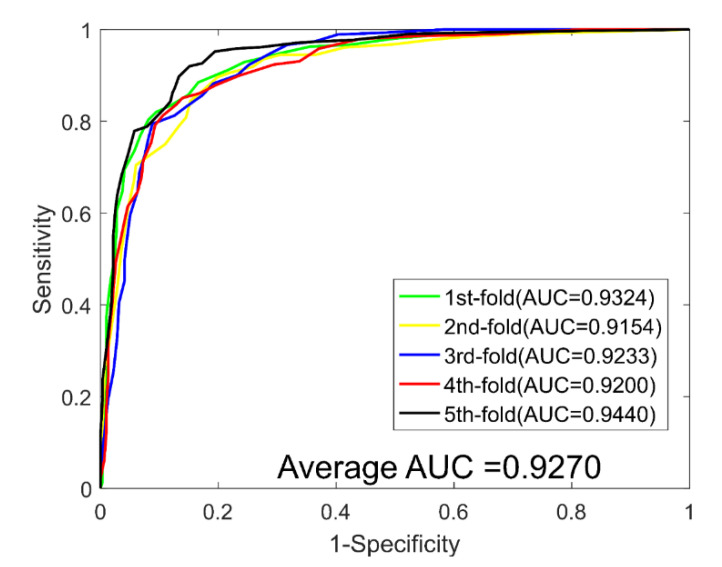
ROC curves yielded for RoF on the Ion Channel dataset.

**Figure 5:**
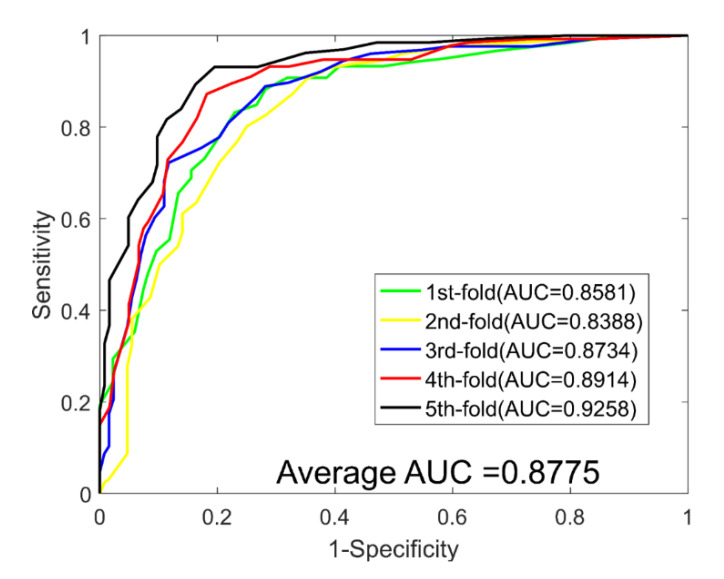
ROC curves yielded for RoF on the GPCRs dataset.

**Figure 6:**
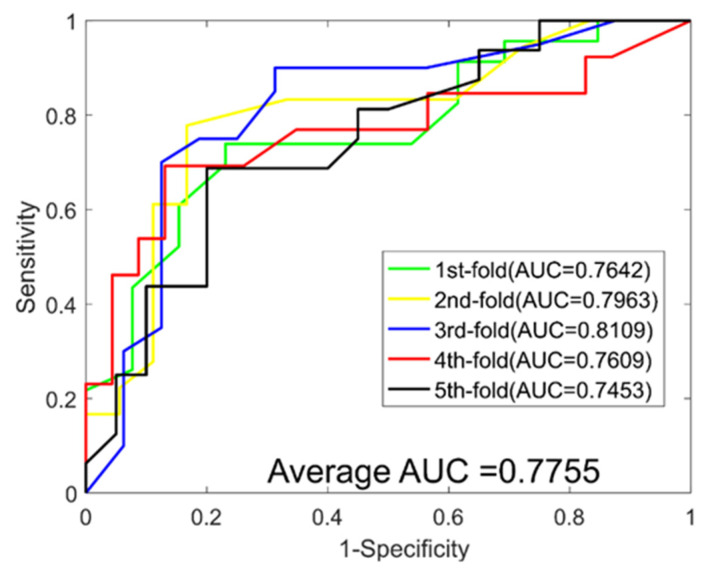
ROC curves yielded for RoF on the Nuclear Receptors dataset.

When predicting DTIs for the *Enzyme* dataset, we yielded average ACC., PR., Sen., MCC, and AUC values of 89.21%, 90.77%, 87.30%, 80.73%, and 0.9498, with corresponding standard deviations of 0.69%, 1.40%, 1.62%, 1.09%, and 0.0059, respectively. When predicting DTIs for the *Ion Channel* dataset, we yielded average ACC., PR., Sen., MCC, and AUC values of 85.49%, 85.58%, 85.40%, 75.12%, and 0.9270, with corresponding standard deviations of 1.18%, 1.32%, 3.54%, 1.73%, and 0.0113, respectively. When predicting DTIs for the *GPCRs* dataset, we yielded average ACC., PR., Sen., MCC, and AUC values of 81.02%, 81.51%, 80.38%, 69.42% and 0.8775, with corresponding standard deviations of 3.77%, 2.90%, 4.47%, 4.76% and 0.0332, respectively. When predicting DTIs for the *NRs* dataset, we yielded average ACC., PR., Sen., MCC, and AUC values of 74.44%, 72.31%, 78.17%, 61.23%, and 0.7755, with corresponding standard deviations of 5.34%, 7.29%, 10.64%, 5.75%, and 0.0271, respectively. The ROC curves of the RoF classifier obtained for the four datasets are shown in Figure 3, Figure 4, Figure 5 and Figure 6.

To prove that the predictive performance of our model is not dependent on the selection of negative samples, we applied our method to five different GPCRs negative samples, which were randomly selected from the non-interacting drug–target pairs. The predictive results of the five different negative samples are listed in Table 5. It can be observed that the experimental results of these five samples were not significantly different. The average ACC., PR., Sen., MCC, and AUC values are higher than 81%, 82%, 79%, 69% and 0.88, respectively. These results further indicate that our method for constructing the negative samples in this work is effective for predicting potential DTIs. The remarkable prediction performance can be attributed to the robust feature descriptors and powerful RoF classifier. The application of DTCWT to extract feature vectors is novel and effective. As a sequence encoding method, PSSM can retain the useful information of amino acid sequences. The excellent results suggested that the RoF algorithm is suitable for detecting potential drug–target proteins.

### 2.4. Comparison Results between LPQ-Based Model and the Proposed Method

Many describers have been introduced to detect DTIs, with local phase quantization (LPQ) [27] being one of the most popular algorithms. To verify the performance of the DTCWT descriptor, we compared it with the LPQ method. The cross-validation results of the LPQ descriptor combined with the RoF classifier are summarized in Table 6. It can be observed that the proposed approach generated the best results in terms of ACC, PR, MCC, and AUC values. For the sake of consistency, the same parameters were used in the comparison experiment. From the comparison results, we can observe that the DTCWT descriptor combined with the RoF classifier can improve the prediction performance of the model. The detailed 5-fold CV results performed by the LPQ algorithm on the four datasets are summarized in the Supplementary Materials, Tables S1–S4.

### 2.5. Comparison with SVM and KNN Classifier

Various machine learning algorithms have previously been used to identify DTIs [28,29]. To further evaluate the predictive capacity of our method, we used the same feature descriptors in the SVM and KNN classifiers and compared the predictive performance using the same four datasets. The main idea of the SVM algorithm is that it can perform both linear classification and non-linear classification problems. KNN is a supervised machine learning technique which can solve the classification task. The LIBSVM tool [30] was used in this paper to train the SVM model. There are two parameters of SVM that need to be optimized: *c* (penalty parameters) and *g* (kernel function parameters). The parameters *c* and *g* from the SVM classifier were optimized by a grid search method, with *c* values from 1 to 25 and *g* values from 0.1 to 5. In the experiments for the Enzyme and Ion Channel datasets, we set *c* = 7, *g* = 0.2 and *c* = 3, *g* = 4, respectively. When exploring the proposed method for the *GPCRs* and *NRs* datasets, we set *c* = 7, *g* = 1.3 and *c* = 23, *g* = 0.1, respectively. The KNN model needs to choose the neighbor *k* and distance measuring function. Here, we optimized K from 1 to 10 to train the KNN model. In this paper, K has been set as 5 and the distance measuring function as L1.

Table 7 lists all the experimental results of RoF, SVM, and KNN models on the four DTIs datasets. From these results, we can see that our method achieves better prediction results than SVM- and KNN-based methods. For example, the AUC gaps between SVM and RoF on the four datasets were 0.1486, 0.1587, 0.2123, and 0.1535, respectively. Similarly, the ACC gaps between KNN and RoF were 8.68%, 6.47%, 17.16%, and 26.11%, respectively. The ROC curves and comparison results yielded by the SVM and KNN models are shown in the Supplementary Materials, Figures S1–S5.

### 2.6. Comparison with Different Methods on the Same Dataset

In recent years, many different kinds of excellent computational approaches have been put forward to predict DTIs. To further confirm the effectiveness of our method, we compared it with some previous prediction studies, which used the same benchmark datasets, including Yamanishi et al. [31], KBMF2K [32], MLCLE [33], AM-PSSM [34], SIMCOMP [35], DBSI [36], and NETCBP [37]. The average AUC values of these approaches are summarized in Table 8. It can be observed that our method performed better than other methods on the Enzyme, Ion Channel, and GPCRs datasets. However, it did not work very well on he NRs dataset, perhaps because the NRs dataset was too small to optimally train the RoF model.

### 2.7. Performance on the Independent Dataset

To demonstrate the generalizability of our model, we performed it on an independent dataset. The Enzyme dataset was used as the training set and the Drugbank-approved dataset was employed as the testing dataset. For fairness, we set the same parameters as for the RoF model (*K* = 30, *L* = 17). When applying our model on the Drugbank-approved dataset, it yielded a high accuracy of 72.37%, PR. of 69.23%, Sen. of 74.46%, MCC of 59.94%, and AUC of 0.7833. The predictive results on the independent dataset further indicate that our method is useful for predicting unknown DTI pairs.

## 3. Materials and Methods

### 3.1. Data Collection

In this work, we selected four DTI datasets: Enzyme, Ion Channel, GPCRs and Nuclear Receptors (NRs). These data can be collected from BRENDA [38], KEGG [39], SuperTarget database [40], and DrugBank [41]. The numbers of drug compounds, target proteins, and known interactions are summarized in Table 9. We constructed a bipartite graph to present the relations between drugs and proteins, where the nodes represent the target proteins or drug compounds, and the links represent the interactions between them. Here, we set the Ion Channel dataset as an example, for which the total number of interactions is 42840 (204 × 210) in the corresponding bipartite. However, only 1476 pairs have been shown to have interactions. Thus, the possible number of negative Ion Channel DTI pairs is 41364 (42840−1476), which is significantly more than the positive samples. To deal with this bias problem, we randomly collectedly 1476 non-interacting DTI pairs as the negative samples. The negative samples that we obtained from this method may contain some truly interacting pairs. However, given the size of the DTI datasets, the probability of this situation is very small.

We also used a dataset called Drugbank-approved [42] as the independent dataset to further verify the predictive ability of our model. The drugs and proteins in this dataset are all approved by the FDA and DrugBank database [41]. After removing the non-existing drugs and proteins, we obtained 1555 drugs, 1591 target proteins, and 5831 interactions.

### 3.2. Characterization of Drug Molecules

From previous research, many descriptors have been proposed to represent the properties of drug molecules, such as the topological, geometric, constitutional, and quantum chemical descriptors. Recently, some studies found that molecular substructure fingerprints can be used to represent drug compound structures [43]. By encoding the drugs as Boolean substructure vectors, the substructure fingerprints can directly indicate whether each compound has a specific chemical substructure of the drug molecule. It proves that after being separated into fragments, its substructure remains. In a binary fingerprint vector, each bit position will correspond to a specific substructure. If the corresponding substructure of a given drug molecule is present, the corresponding bit will be set to 1; otherwise, it will be assigned to 0. In this way, the complex structures of drug molecules can be represented by the substructure fingerprints. Although the fingerprint splits the whole molecule into many fragments, it can still provide structural information for drug molecules. Moreover, substructure fingerprints do not need 3D structural data for the target, so it will not cause error accumulation.

The substructure fingerprint sets employed in this work were downloaded from the *PubChem* System (available at https://pubchem.ncbi.nlm.nih.gov/, accessed on 4 June 2009). It defines 881 chemical substructures, which have each been assigned to a specific site. Therefore, each drug molecule feature has been transformed into a binary vector of 881 dimensions.

### 3.3. Representation of Target Proteins

The position-specific scoring matrix (PSSM) [44] was proposed for testing the distantly related proteins. In recent years, PSSM has been widely used for mining the evolutionary information of protein sequences [45]. The PSSM is a P×20 matrix. The number of amino acids in the proteins is represented by *P*, and the naive amino acids are represented by 20 columns. Supposing $L=\left\{\varphi \left(i,j\right):i=1\cdots P,j=1\cdots 20\right\}$, then the following is a summary of each matrix:

(5) $$L=\left[\begin{array}{llllll} \varphi_{1,1} & \varphi_{1,\;2} & \cdots & \varphi_{1,j} & \cdots & \varphi_{1,\;20}\\ \varphi_{2,1} & \varphi_{2,\;2} & \cdots & \varphi_{2,j} & \cdots & \varphi_{2,\;20}\\ \vdots & \vdots & & \vdots & \vdots & \\ \varphi_{i,1} & \varphi_{i,\;2} & \cdots & \varphi_{i,j} & \cdots & \varphi_{i,\;20}\\ \vdots & \vdots & & \vdots & \vdots & \\ \varphi_{p,1} & \varphi_{p,\;2} & \cdots & \varphi_{p,j} & \cdots & \varphi_{p,\;20} \end{array}\right]$$

where $\varphi_{i,j}$ in the *i*^th^ row of PSSM indicates the probability of the *i*^th^ residue being mutated into the *j*^th^ native amino acid.

In this article, we used the position-specific iterated BLAST (PSI-BLAST) [46] tool, which was against the database of SwissProt, to generate the PSSM for the purpose of extracting evolutionary information. To obtain high homologous sequences, the expectation value (*e-value*) was set to 0.001, the number of iterations was set to 3, and other parameters were maintained as the default values [47]. The SwissProt database and PSI-BLAST can be freely obtained from http://blast.ncbi.nlm.nih.gov/Blast.cgi (accessed on 1 January 2001).

### 3.4. Feature Extraction Method

The dual-tree complex wavelet transform (DTCWT) [48] is an enhanced version of the discrete wavelet transform (DWT) [49]. It was developed to help improve the directional selectivity impaired by DWT. In addition, it compensates for the fact that DWT has a large computation volume and high complexity. Unlike conventional DWT, DTCWT is constructed by two real DWTs [50]. The first DWT is used to generate the real part of the transform, while the second DWT generates the imaginary part.

**Figure 7:**
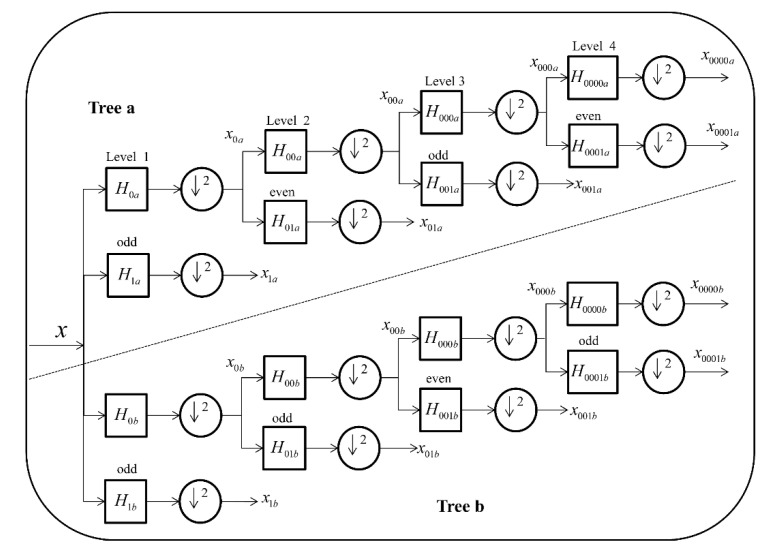
DTCWT is implemented employing two wavelet filter banks in parallel.

The DTCWT addressed the disadvantages of DWT regarding shift-invariant problems and directional selectivity in two or more dimensions. The directional selectivity of DTCWT can be yielded by the wavelets, which are approximately analytic. It can produce six directionally selective sub-bands (±15°, ±45° and ±75°), with (*R*) and (*I*) describing the real and imaginary parts, respectively. The flowchart of the DTCWT algorithm is shown in Figure 7. In the first stage, the filters can be denoted as $h_{i}(n)$ and $g_{i}(n)$. The first filter bank can be represented by $H_{new}^{\left(p\right)}(e^{jw})$ and second filter bank can be represented by Hnew(p)′(ejw). In this way, we obtain the following corollary:

**Corollary 1**. Suppose we have CQF pairs $\left\{h_{0}(m),h_{1}(m)\right\},\left\{h_{0}{}^{\prime }(m),h_{1}{}^{\prime }(m)\right\}$, for p>1.

(6) Hnew(p)(ejw)=HHnew(p)′(ejw)

if and only if

(7) $$h_{0}{}^{{}^{\prime }(1)}(m)=h_{0}^{\left(1\right)}(m-1)$$

A 2D image $F\left(a,b\right)$ is formed by 2D DTCWT over the complex scaling function and a series of expansion and translation of six wavelet functions $\alpha_{j,l}^{\theta }$, that is,

(8) $$F\left(a,b\right)=\sum_{l\in Z^{2}}s_{j_{0},l^{\phi }j_{0}’}l^{\left(a,b\right)}+\sum_{\theta \in \Theta }\sum_{j\geq j_{0}}\sum_{l\in Z^{2}}c_{j}^{\theta },l^{\alpha_{j}^{\theta }},l^{\left(a,b\right)}$$

The directionality of the complex wavelet function is provided by $\theta \in \Theta =\left\{\pm 15{}^{\circ},\pm 45{}^{\circ},\pm 75{}^{\circ}\right\}$. That said, at each decomposition level, $F\left(a,b\right)$ is decomposed by DTCWT into a low-pass sub-band and six complex valued high-pass sub-bands, and each high-pass sub-bands corresponds to a specific direction θ. In this experiment, after a PSSM matrix was performed via DTCWT, each target protein sequence was defined as a 256-dimensional feature vector.

### 3.5. Rotation Forest Classifier (RoF)

Rotation Forest (RoF) is an effective and powerful ensemble learning method which was first proposed by Rodriguez [51]. The main contribution of RoF is to establish the ensemble classifiers that can obtain a balance between diversity and accuracy. In this algorithm, the attribute set of samples is first randomly divided, and each subset is transformed by a linear transformation to increase the diversity of samples. Then, the transformed subsets are fed into different decision trees, and the final classification results can be aggregated from the votes of all trees in the forest.

Suppose that $\left\{q_{i},p_{i}\right\}$ contains *T* samples, of which $q_{i}=\left(q_{i1},q_{i2},q_{i3},\cdots ,q_{iL}\right)$ is an *L*-dimensional feature vector. Let *Z* represent the training sample set containing *T* training samples, forming a matrix of T×L. Let *U* represent the feature set and *M* the label set. Assume the number of decision trees is *S*, then the decision trees can be denoted as $D_{1},D_{2},D_{3},\cdots ,D_{S}$. The rotation forest algorithm is implemented as follows.

(1) Choose a suitable parameter *M* for which *U* can be randomly split into *M* disjointed subsets, with the number of features contained in the feature subset being equal to *L*/*M*.
(2) Let $U_{i,j}$ represent the *j*^th^ feature subset and use it to train the classifier $D_{i}$. The sample subset $Z_{i,j}^{\prime }$ is constructed by a non-empty subset, which is randomly selected from a certain proportion.
(3) Apply PCA [52] on $Z_{i,j}^{\prime }$ to order the coefficients stored in matrix $\lambda_{i,j}$.
(4) The coefficients obtained from the matrix $\lambda_{i,j}$ are used to construct a sparse rotation matrix $\varphi_{i}$, which can be defined as follows:
  (9) $$\varphi_{i}=\left[\begin{array}{cccc} a_{i,1}^{\left(1\right)},\cdots ,a_{i,1}^{\left(S_{1}\right)} & 0 & \cdots & 0\\ 0 & a_{i,1}^{\left(1\right)},\cdots ,a_{i,1}^{\left(S_{1}\right)} & \cdots & 0\\ \cdots & \vdots & \ddots & \vdots \\ 0 & 0 & \cdots & a_{i,1}^{\left(1\right)},\cdots ,a_{i,1}^{\left(S_{M}\right)} \end{array}\right]$$

During the prediction process, given a test sample *g* that is generated by the classifier $D_{i}$ of $R_{i,j}(Z\varphi_{i}^{a})$, which is introduced to indicate that *g* belongs to class $p_{i}$. Then, the class of confidence is calculated via the average combination; the formula can be expressed follows:

(10) $$V_{j}(g)=\frac{1}{S}\sum_{i=1}^{S}R_{i,j}(Z\varphi_{i}^{a})$$

Then, assign the category with the largest $V_{j}(g)$ value to *g*.

## 4. Case Study

To further demonstrate the generality of the proposed method, we applied our method on two real-life drug–target pairs; the drug was *Flurbiprofen* and two target proteins were *prostaglandin-endoperoxide synthase 1-type* and *inhibitor of nuclear factor kappa-B kinase subunit epsilon*. The lengths of the two proteins are 599 and 716, respectively. Our method predicted that the drug *Flurbiprofen* would interact with the target protein *prostaglandin-endoperoxide synthase 1-type* with a probability score of 0.96, and would not interacted with the target protein *inhibitor of nuclear factor kappa-B kinase subunit epsilon* with a probability score of 0.08. The interacting drug–target pairs have been confirmed by the KEGG database. The experimental results of these two real-life drug–target pairs further indicates that the proposed model is effective for predicting potential DTIs.

## 5. Conclusions

In this article, we developed a novel computational method for identifying DTIs using information regarding target protein sequences and the substructure fingerprints of drug molecules. It combines the position-specific scoring matrix (PSSM), dual-tree complex wavelet transform (DTCWT), and Rotation Forest (RoF). In order to evaluate the prediction performance of the proposed method, we performed tests on four datasets (Enzyme, Ion Channel, GPCRs, and Nuclear Receptors) by adopting a 5-fold cross validation (*CV*). The proposed approach obtained high average accuracies of 89.21%, 85.49%, 81.02%, and 74.44%, respectively. To verify the predictive capacity of our method, we compared it with the SVM and KNN algorithms and some existing studies. The experimental results on the independent dataset further demonstrated that our model can be used as a valuable tool to predict potential drug–target interactions. In the future, we need to find more efficient feature extraction methods and reduce the computational complexity for DTI prediction.

## Figures and Tables

**Table 1:** 5-fold *CV* results yielded when predicting DTIs for the Enzyme dataset.

Test Set	ACC. (%)	PR. (%)	Sen. (%)	MCC (%)	AUC
1	89.40	89.80	87.88	81.00	0.9452
2	88.72	89.02	88.72	79.98	0.9516
3	88.80	90.84	85.96	80.07	0.9499
4	88.80	91.68	85.22	80.06	0.9435
5	90.34	92.49	88.71	82.53	0.9585
**Average**	89.21 ± 0.69	90.77 ± 1.40	87.30 ± 1.62	80.73 ± 1.09	0.9498 ± 0.0059

**Table 2:** 5-fold *CV* results yielded when predicting DTIs for the Ion Channel dataset.

Test Set	ACC. (%)	PR. (%)	Sen. (%)	MCC (%)	AUC
1	85.08	85.08	85.08	74.62	0.9324
2	84.41	86.26	80.14	73.49	0.9154
3	84.92	87.06	84.59	74.27	0.9233
4	85.59	83.59	89.40	75.22	0.9200
5	87.46	85.92	87.77	78.01	0.9440
**Average**	85.49 ± 1.18	85.58 ± 1.32	85.40 ± 3.54	75.12 ± 1.73	0.9270 ± 0.0113

**Table 3:** 5-fold *CV* results yielded when predicting DTIs for the GPCRs dataset.

Test Set	ACC. (%)	PR. (%)	Sen. (%)	MCC (%)	AUC
1	79.13	81.06	79.26	66.88	0.8581
2	76.77	77.60	75.78	64.33	0.8388
3	79.53	80.16	78.91	67.44	0.8734
4	83.46	84.35	80.17	72.24	0.8914
5	86.22	84.38	87.80	76.23	0.9258
**Average**	81.02 ± 3.77	81.51 ± 2.90	80.38 ± 4.47	69.42 ± 4.76	0.8775 ± 0.0332

**Table 4:** 5-fold *CV* results yielded when predicting DTIs for the NRs dataset.

Test Set	ACC. (%)	PR. (%)	Sen. (%)	MCC (%)	AUC
1	72.22	60.00	59.23	57.67	0.7642
2	77.78	72.73	88.89	64.58	0.7963
3	80.56	73.69	87.50	68.43	0.8109
4	66.67	78.95	65.22	53.74	0.7609
5	75.00	76.19	80.00	61.73	0.7453
**Average**	74.44 ± 5.34	72.31 ± 7.29	78.17 ± 10.64	61.23 ± 5.75	0.7755 ± 0.0271

**Table 5:** 5-fold CV results obtained from our method from five different negative samples from the GPCRs dataset.

Negative Samples	ACC. (%)	PR. (%)	Sen. (%)	MCC (%)	AUC
Sample 1	81.73 ± 2.18	82.47 ± 2.48	80.65 ± 3.93	70.03 ± 2.94	0.8882 ± 0.0126
Sample 2	81.13 ± 1.80	83.04 ± 2.49	80.59 ± 4.64	70.51 ± 2.36	0.8904 ± 0.0167
Sample 3	81.97 ± 1.06	82.19 ± 1.79	81.59 ± 3.39	70.29 ± 1.48	0.8876 ± 0.0046
Sample 4	81.10 ± 1.18	82.04 ± 1.61	79.53 ± 4.17	69.18 ± 1.65	0.8881 ± 0.0162
Sample 5	81.57 ± 1.53	82.79 ± 2.66	81.23 ± 4.59	69.76 ± 2.06	0.8900 ± 0.0124

**Table 6:** Comparison of experimental results of LPQ and DTCWT with Rotation Forest classifier on four golden standard datasets.

Dataset	Descriptor	ACC. (%)	PR. (%)	Sen. (%)	MCC (%)	AUC
Enzyme	LPQ	87.45 ± 3.87	88.62 ± 5.32	86.20 ± 2.02	78.24 ± 5.49	0.9329 ± 0.0358
	DTCWT	89.21 ± 0.69	90.77 ± 1.40	87.30 ± 1.62	80.73 ± 1.09	0.9498 ± 0.0059
Ion Channel	LPQ	84.07 ± 3.59	83.10 ± 3.98	85.49 ± 4.85	73.43 ± 5.03	0.9008 ± 0.0404
	DTCWT	85.49 ± 1.18	85.58 ± 1.32	85.40 ± 3.54	75.12 ± 1.73	0.9270 ± 0.0113
GPCRs	LPQ	79.61 ± 4.26	78.69 ± 6.04	81.07 ± 3.76	67.71 ± 4.69	0.8474 ± 0.0402
	DTCWT	81.02 ± 3.77	81.51 ± 2.90	80.38 ± 4.47	69.42 ± 4.76	0.8775 ± 0.0332
NRs	LPQ	71.11 ± 3.17	70.03 ± 10.36	75.55 ± 13.97	56.53 ± 3.54	0.7403 ± 0.0667
	DTCWT	74.44 ± 5.34	72.31 ± 7.29	78.17 ± 10.64	61.23 ± 5.75	0.7755 ± 0.0271

**Table 7:** Comparing results from RoF with SVM and KNN models on the four datasets.

Dataset	Model	ACC. (%)	PR. (%)	Sen. (%)	MCC (%)	AUC
Enzyme	SVM	77.25 ± 0.73	79.84 ± 1.31	72.86 ± 2.45	64.67 ± 0.89	0.8012 ± 0.0191
	KNN	80.53 ± 1.85	77.42 ± 2.20	86.06 ± 3.17	68.46 ± 2.17	0.8050 ± 0.0168
	RoF	89.21 ± 0.69	90.77 ± 1.40	87.30 ± 1.62	80.73 ± 1.09	0.9498 ± 0.0059
Ion Channel	SVM	71.08 ± 1.93	70.28 ± 1.56	73.21 ± 4.58	58.77 ± 1.61	0.7683 ± 0.0224
	KNN	79.02 ± 2.28	75.11 ± 3.60	86.72 ± 3.89	66.41 ± 2.68	0.7901 ± 0.0204
	RoF	85.49 ± 1.18	85.58 ± 1.32	85.40 ± 3.54	75.12 ± 1.73	0.9270 ± 0.0113
GPCRs	SVM	61.65 ± 2.44	62.26 ± 4.33	59.53 ± 1.50	52.72 ± 1.09	0.6652 ± 0.0252
	KNN	63.86 ± 3.83	61.47 ± 3.05	74.65 ± 2.21	52.86 ± 2.61	0.6379 ± 0.0408
	RoF	81.02 ± 3.77	81.51 ± 2.90	80.38 ± 4.47	69.42 ± 4.76	0.8775 ± 0.0332
NRs	SVM	62.22 ± 5.41	61.99 ± 11.70	60.90 ± 9.47	52.22 ± 3.51	0.6220 ± 0.0611
	KNN	48.33 ± 6.97	48.65 ± 9.71	46.68 ± 2.26	49.58 ± 0.77	0.4775 ± 0.0728
	RoF	74.44 ± 5.34	72.31 ± 7.29	78.17 ± 10.64	61.23 ± 5.75	0.7755 ± 0.0271

**Table 8:** Comparison results of different approaches on the four datasets in terms of AUC values.

Method	Enzyme	GPCRs	Ion Channel	NRs
Yamanishi et al.	0.845	0.812	0.731	0.830
KBMF2K	0.832	0.857	0.799	0.824
MLCLE	0.842	0.850	0.795	0.790
AM-PSSM	0.843	0.839	0.722	0.767
SIMCOMP	0.863	0.867	0.776	0.856
DBSI	0.8075	0.8022	0.8029	0.7578
NETCBP	0.8251	0.8235	0.8034	0.8394
Our Method	0.9498	0.8775	0.9270	0.7755

**Table 9:** Statistical data information for the five datasets.

Dataset	Drug	Target Protein	Interactions
Enzyme	445	664	2926
Ion Channel	210	204	1476
NRs	54	26	90
GPCRs	223	95	635
Drugbank-approved	1555	1591	5831

## Data Availability

All the data are available at https://github.com/jie-pan111/prediction_of_DTIs (1 September 2021) and protein sequence data are available at http://web.kuicr.kyoto-u.ac.jp/supp/yoshi/drugtarget/, accessed on 1 September 2021.

## Supplementary Materials

The following are available online, Table S1: 5-fold CV results achieved by LPQ-based method on Enzyme dataset, Table S2: 5-fold CV results achieved by LPQ-based method on Ion Channel dataset, Table S3: 5-fold CV results achieved by LPQ-based method on GPCRs dataset, Table S4: 5-fold CV results achieved by LPQ-based method on Nuclear Receptor dataset, Figure S1: Performance comparisons of five validation metrics for three models: RF (blue bar), SVM (green bar) and KNN (yellow bar). (a) Accuracy. (b) Precision. (c) MCC. (d) AUC, Figure S2: The ROC curves achieved on the Enzyme dataset (5-fold CV). (a) is the ROC curves generated by SVM model. (b) is the ROC curves generated by KNN classifier, Figure S3: The ROC curves achieved on the Ion Channel dataset (5-fold CV). (a) is the ROC curves generated by SVM model. (b) is the ROC curves generated by KNN classifier, Figure S4: The ROC curves achieved on the GPCRs dataset (5-fold CV). (a) is the ROC curves generated by SVM model. (b) is the ROC curves generated by KNN classifier, Figure S5: The ROC curves achieved on the Nuclear Receptors dataset (5-fold CV). (a) is the ROC curves of SVM model. (b) is the ROC curves of KNN classifier.

## Sample Availability

Samples of the compounds are available from the authors.
