# Supplementary material for: Prediction of Drug–Target Interactions by Combining Dual-Tree Complex Wavelet Transform with Ensemble Learning Method
Source: Molecules. 2021 Sep 3;26(17):5359. doi: 10.3390/molecules26175359 (PMC8433937; doi:10.3390/molecules26175359)
Supplement: Supplementary file 1 [file molecules-26-05359-s001.zip › molecules-1364063-supplementary.pdf]

Table S1. 5-fold CV results achieved by LPQ-based method on ***Enzyme*** dataset.

Test set	ACC. (%)	PR. (%)	Sen. (%)	MCC (%)	AUC (%)
1	89.15	90.86	86.19	80.57	94.57
2	88.29	89.42	87.48	79.32	94.17
3	90.77	92.83	88.80	83.23	96.05
4	80.77	79.36	83.78	68.86	87.01
5	88.29	90.64	84.76	79.24	94.63
Average	87.45±3.87	88.62±5.32	86.20±2.02	78.24±5.49	93.29±3.58

Table S2. 5-fold CV results achieved by LPQ-based method on ***Ion Channel*** dataset.

Test set	ACC. (%)	PR. (%)	Sen. (%)	MCC (%)	AUC (%)
1	86.61	83.90	90.94	76.70	92.97
2	84.58	84.87	85.15	73.89	90.48
3	84.75	81.69	87.00	74.10	91.18
4	77.29	77.18	77.70	64.89	83.08
5	87.12	87.84	86.67	77.55	92.67
Average	84.07±3.95	83.10±3.98	85.49±4.85	73.43±5.03	90.08±4.04

Table S3. 5-fold CV results achieved by LPQ-based method on ***GPCRs*** dataset.

Test set	ACC. (%)	PR. (%)	Sen. (%)	MCC (%)	AUC (%)
1	79.53	75.40	81.90	67.31	87.82
2	83.46	81.40	85.37	72.38	88.48
3	81.89	84.17	82.98	70.02	85.16
4	72.44	69.70	75.41	60.01	78.40
5	80.71	82.81	79.70	68.82	83.86
Average	79.61±4.26	78.68±6.04	81.07±3.76	67.71±4.69	84.74±4.02

Table S4. 5-fold CV results achieved by LPQ-based method on ***Nuclear Receptor*** dataset.

Test set	ACC. (%)	PR. (%)	Sen. (%)	MCC (%)	AUC (%)
1	72.22	76.47	68.42	59.75	72.45
2	75.00	76.00	86.36	58.41	64.77
3	72.22	60.00	69.23	57.67	82.94
4	69.44	80.00	60.00	56.69	72.97
5	66.67	57.69	93.75	50.62	77.03
Average	71.11±3.17	70.03±10.36	75.55±13.97	56.63±3.54	74.03±6.67

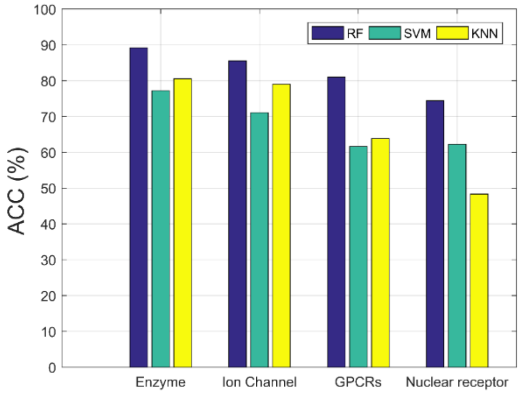

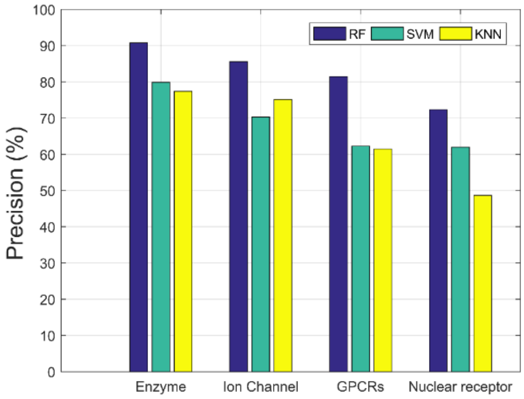

(**a**) (**b**)

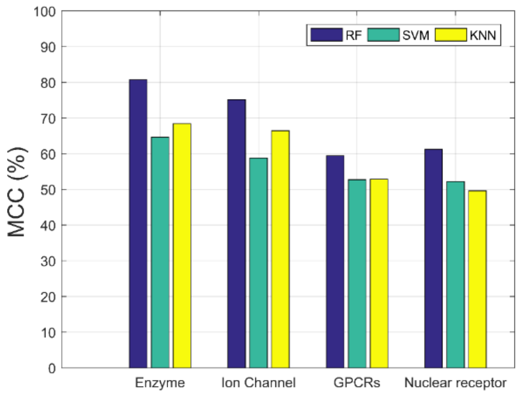

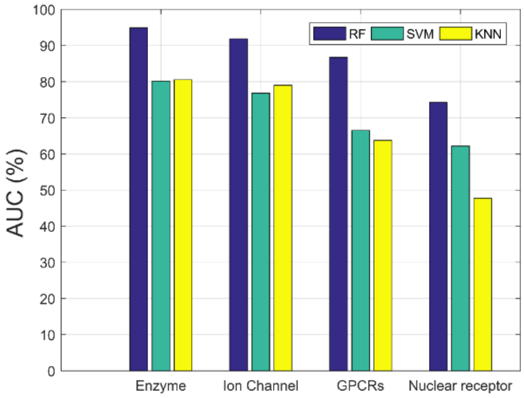

(c) (d)

Figure S1. Performance comparisons of five validation metrics for three models: RF (blue bar), SVM (green bar) and KNN (yellow bar). (a) Accuracy. (b) Precision. (c) MCC. (d) AUC.

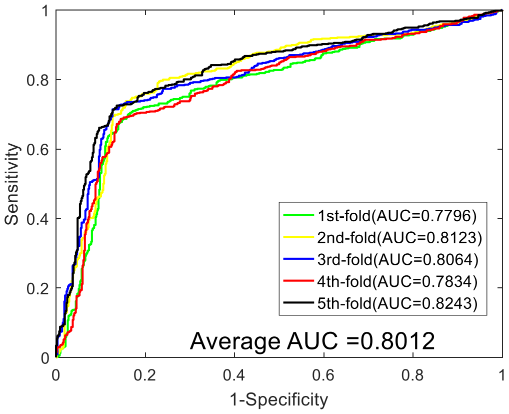

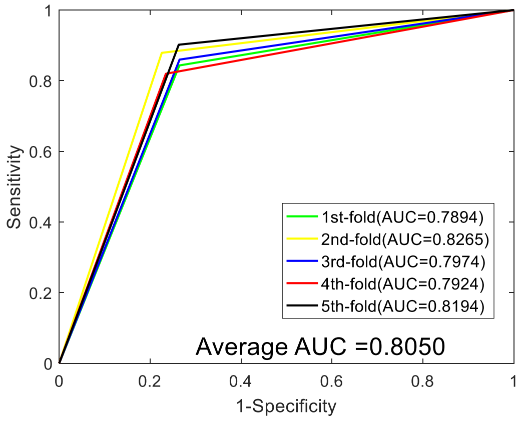

(**a**) (**b**)

Figure S2. The ROC curves achieved on the ***Enzyme*** dataset (5-fold *CV*). (a) is the ROC curves generated by SVM model. (b) is the ROC curves generated by KNN classifier

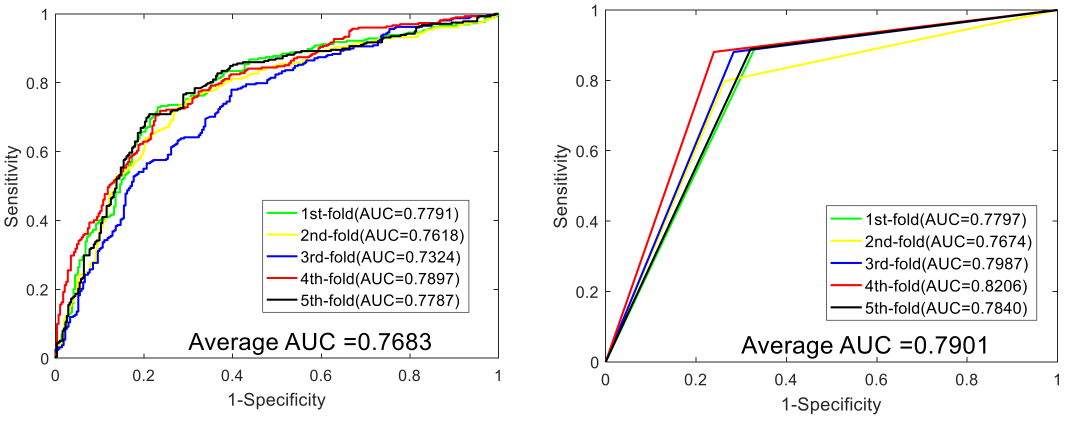

(a) **(b)**

Figure S3. The ROC curves achieved on the ***Ion Channel*** dataset (5-fold *CV*). (a) is the ROC curves generated by SVM model. (b) is the ROC curves generated by KNN classifier

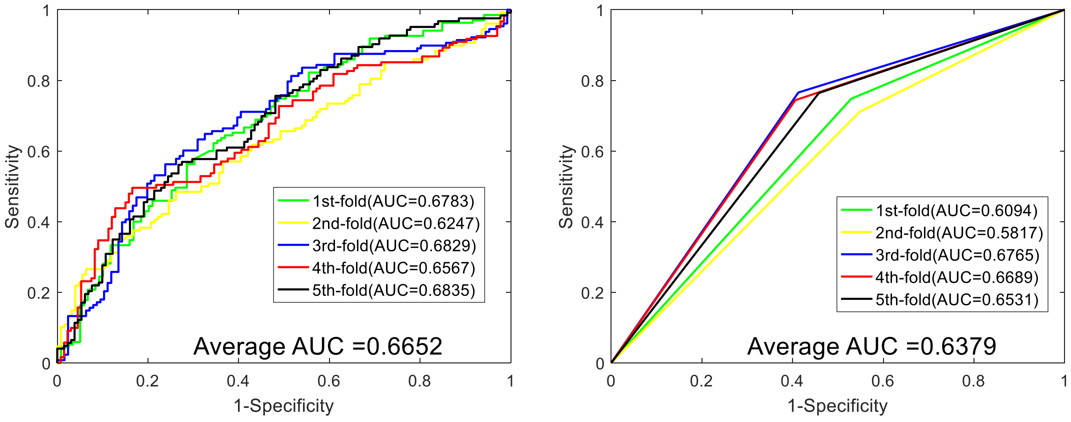

(a) **(b)**

Figure S4. The ROC curves achieved on the ***GPCRs*** dataset (5-fold *CV*). (a) is the ROC curves generated by SVM model. (b) is the ROC curves generated by KNN classifier

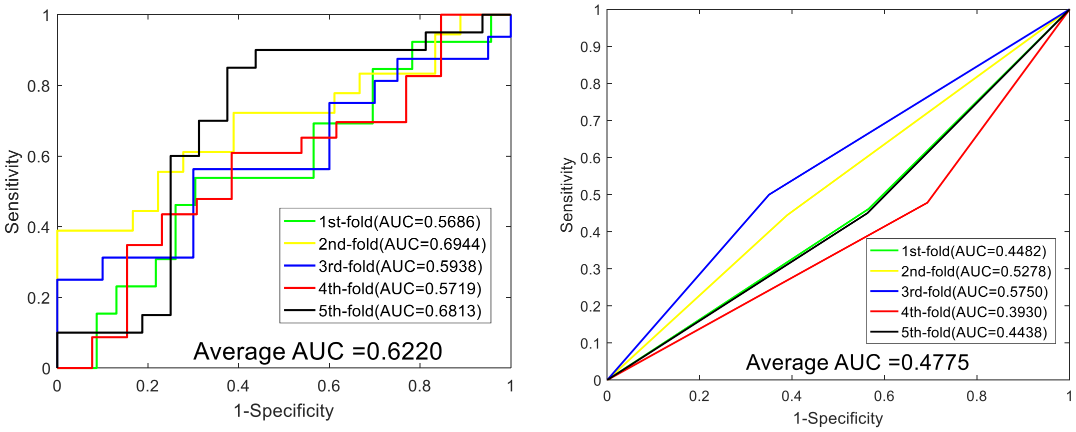

(a) **(b)**

Figure S5. The ROC curves achieved on the ***Nuclear Receptors*** dataset (5-fold *CV*). (a) is the ROC curves of SVM model. (b) is the ROC curves of KNN classifier
